# Epidemiology of chicken anemia virus in Central African Republic and Cameroon

**DOI:** 10.1186/1743-422X-9-189

**Published:** 2012-09-08

**Authors:** Chantal J Snoeck, Giscard F Komoyo, Bonya P Mbee, Emmanuel Nakouné, Alain Le Faou, Mbah P Okwen, Claude P Muller

**Affiliations:** 1 Institute of Immunology, Centre de Recherche Public de la Santé/National Public Health Laboratory, 20A rue Auguste Lumière, L-1950, Luxembourg, Luxembourg; 2 Institut Pasteur de Bangui, Bangui, Central African Republic; 3 District Hospital Bali, North West Regional Delegation of Public Health, Bamenda, Cameroon; 4 Laboratoire de Virologie Hôpital de Brabois Adultes, CHU de Nancy, 54511, Vandoeuvre-lès-Nancy, France

**Keywords:** Chicken anemia virus, Central African Republic, Cameroon, Antibodies, PCR, Phylogeny, Mixed infection

## Abstract

**Background:**

Although chicken anemia virus (CAV) has been detected on all continents, little is known about this virus in sub-Saharan Africa. This study aimed to detect and characterize CAV for the first time in Central African Republic and in Cameroon.

**Results:**

An overall flock seroprevalence of 36.7% was found in Central African Republic during the 2008–2010 period. Virus prevalences were 34.2% (2008), 14.3% (2009) and 10.4% (2010) in Central African Republic and 39% (2007) and 34.9% (2009) in Cameroon. CAV DNA was found in cloacal swabs of 76.9% of seropositive chickens, suggesting that these animals excreted the virus despite antibodies. On the basis of VP1 sequences, most of the strains in Central African Republic and Cameroon belonged to 9 distinct phylogenetic clusters at the nucleotide level and were not intermixed with strains from other continent. Several cases of mixed infections in flocks and individual chickens were identified.

**Conclusions:**

Our results suggest multiple introductions of CAV in each country that later spread and diverged locally. Mixed genotype infections together with the observation of CAV DNA in cloacal samples despite antibodies suggest a suboptimal protection by antibodies or virus persistence.

## Background

Chicken anemia virus (CAV), the only member of the *Gyrovirus* genus in the *Circoviridae* family [1], was first discovered in the late 70’s [2], but a retrospective study revealed that the virus circulated in chickens long before that [3]. Chickens are considered the only natural host of chicken anemia virus, although anti-CAV antibodies have also been detected in Japanese quails [4] but not in other domestic or wild bird species [4,5]. CAV is ubiquitous [6] and the virus seems to be particularly well adapted to its host [7].

The fecal-oral route constitutes probably the main mode of horizontal transmission [8], but CAV was also detected in feather shafts indicating that other modes of dissemination may be possible [9]. Experimental infections via the respiratory tract were also successful [10] but the relevance of such experiments in the field is still unclear. The virus can also be transmitted vertically from infected parents, either the male or female [11,12], irrespective of their antibody status [11,13,14], to their progeny. Seroconversion of specific pathogen-free chickens around the onset of lay without detectable virus in the flock suggested that CAV could be maintained in reproductive organs as a latent or persistent infection with low levels of replication, and become reactivated when the animal reaches sexual maturity [7,11,15].

The virus causes severe anemia, pale bone marrow, thymus atrophy and severe immunosuppression in 2–3 weeks old chickens if they are not protected by maternal antibodies [7]. In older animals infections usually result in subclinical disease that can also lead to economic losses in poultry farms due to reduced weight gain and increased susceptibility to secondary infections [16-19]. Co-infections with Marek’s disease virus or infectious bursal disease virus (IBDV) may lead to a more complex and severe disease [20,21].

Although the poultry sector is an important part of the economy in many African countries, CAV has only been reported without further details from South Africa [22] and more detailed studies were done by us and others in Nigeria [23-27]. In this study, we describe for the first time the presence and the genetic diversity of CAV in Central African Republic (CAF) and in Cameroon (CMR).

## Results

### Seroprevalence

**Figure 1:**
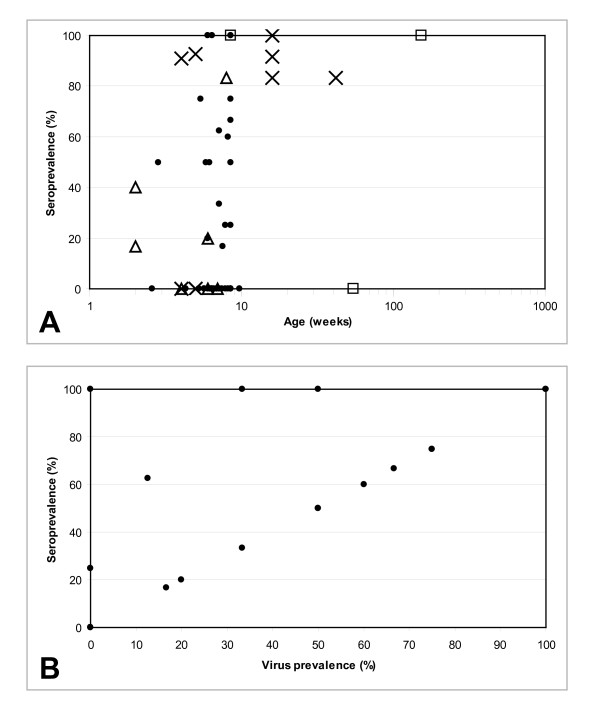
**Anti-CAV antibody seroprevalence in CAF.** (**A**) Age-dependent seroprevalence in broiler flocks () and layer flocks (□) in CAF and in broiler (Δ) or pullet and layer (x) flocks in Nigeria (from Owoade *et al.*[27]). (**B**) Relation between seroprevalence and virus DNA prevalence in chicken flocks in CAF.

In Bangui (CAF), anti-CAV antibodies were found in 147 chicken sera out of the 400 analyzed. In farms, 36.7% (29/79) of the flocks had at least one chicken with anti-CAV antibodies and there was little change throughout the 3 years of sampling: 34.8% (8/23) in 2008, 39.3% (11/28) in 2009 and 35.7% (10/28) in 2010 (Table 1). Four flocks had a seroprevalence below 25%, 5 flocks had a seroprevalence between 25 and 50%, and 20 flocks had a seroprevalence above 50% (Table 1). Most seropositive flocks for which the age was available became seropositive after week 5 (Figure 1A). Several farms were visited 2 or 3 times but no trend could be seen when comparing percentage of seropositive animals between collection time points. In the 2 live bird markets where sera were collected, 69% (68/98) of chickens were seropositive and the seroprevalence in each market was 67.9% (38/56) and 71.4% (30/42).

**Table 1:** Seroprevalence in flocks from Central African Republic

**Year**	**Seroprevalence (%)**					**Total**
	**0**	**> 0 and ≤ 25**	**> 25 and ≤ 50**	**> 50 and ≤ 75**	**> 75 and ≤ 100**	
**2008**	15	1	1	2	4	23
**2009**	17	1	1	1	8	28
**2010**	18	2	3	3	2	28
**Total**	50	4	5	6	14	79

Seroprevalence is expressed as the number of flocks with a seroprevalence included in a percentage range.

### Virus prevalence

In CAF, CAV nucleic acids were detected in 45.8% (11/24) of the flocks in 2008, in 31.7% (19/60) in 2009 and in 15% (9/60) in 2010 (Table 2). Fourteen flocks had a prevalence below 25%, 7 flocks had a prevalence between 25 and 50%, and 18 flocks had a prevalence above 50% (Table 2). Similarly to the seroprevalence data, no trend in the percentage of virus infected animals was observed over the time in farms visited several times. CAV was also detected in 21.2% (42/198) of samples collected in live bird markets and 3 of the 5 markets were infected (prevalence of 27.8% (30/108), 15.5% (11/71) and 9.1% (1/11) over 21 months). For 400 animals, sera and swabs were available and 76.9% (113/147) of the antibody positive animals were PCR positive while all antibody negative animals (253/400) were PCR negatives.

**Table 2:** Virus prevalence in flocks from Central African Republic

**Year**	**Virus prevalence (%)**					**Total**
	**0**	**> 0 and ≤ 25**	**> 25 and ≤ 50**	**> 50 and ≤ 75**	**> 75 and ≤ 100**	
**2008**	13	2	4	3	2	24
**2009**	41	6	3	4	6	60
**2010**	51	6	0	1	2	60
**Total**	105	14	7	8	10	144

Virus prevalence is expressed as the number of flocks with a virus prevalence included in a percentage range.

Overall prevalences of CAV infections of 34.2% (26/76), 14.3% (59/412) and 10.4% (33/316) were found in 2008, 2009 and 2010 respectively in Central African Republic, compared to 39% (112/287) in 2007 and 34.9% (203/582) and 2009 in Cameroon.

In both countries, a total of 433 samples were positive for CAV nucleic acids and were submitted for sequencing.

### Phylogenetic analyses of nucleotide sequences

**Figure 2:**
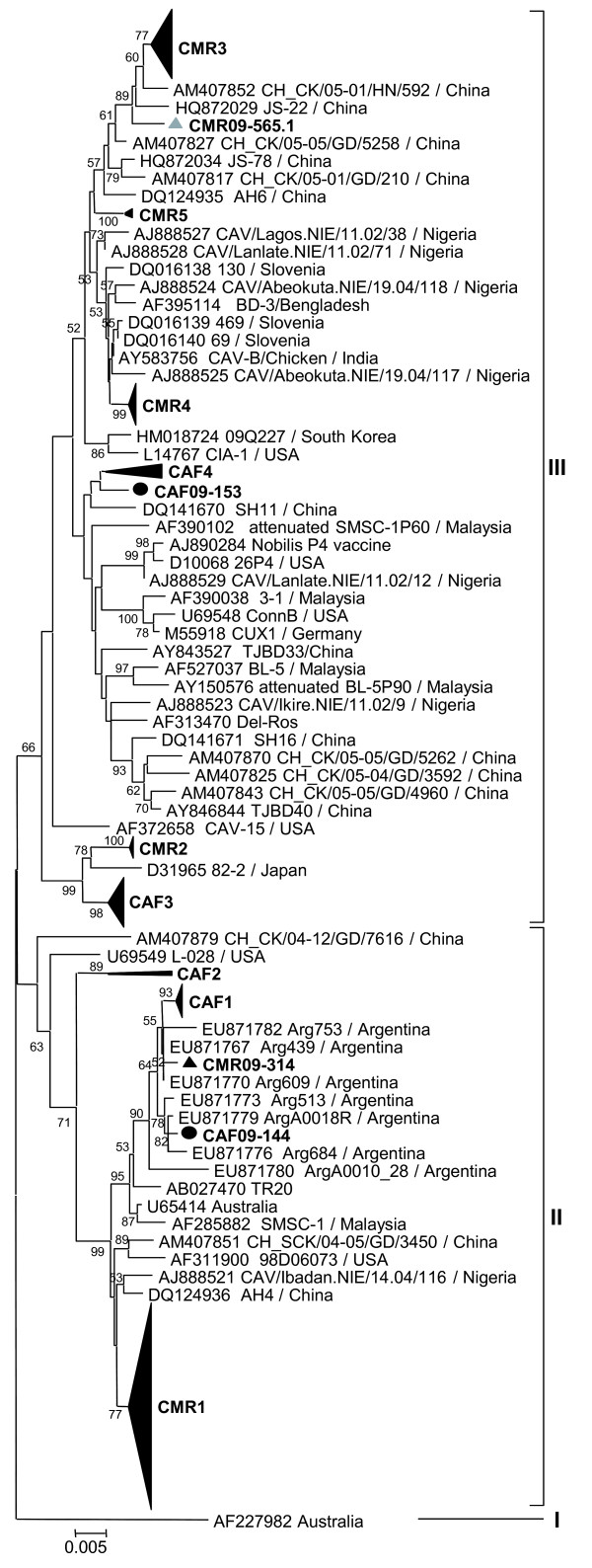
**Phylogenetic analysis of partial VP1 sequences (1281 nucleotides) of 228 CAV strains from Central African Republic () and Cameroon (▴).** Sequences from mixed infected samples are represented by the grey symbols. CAF and CMR clusters are shown as aggregated clusters (CAF1-CAF4; CMR1-CMR5). Only bootstrap values higher than 50% are shown.

**Figure 3:**
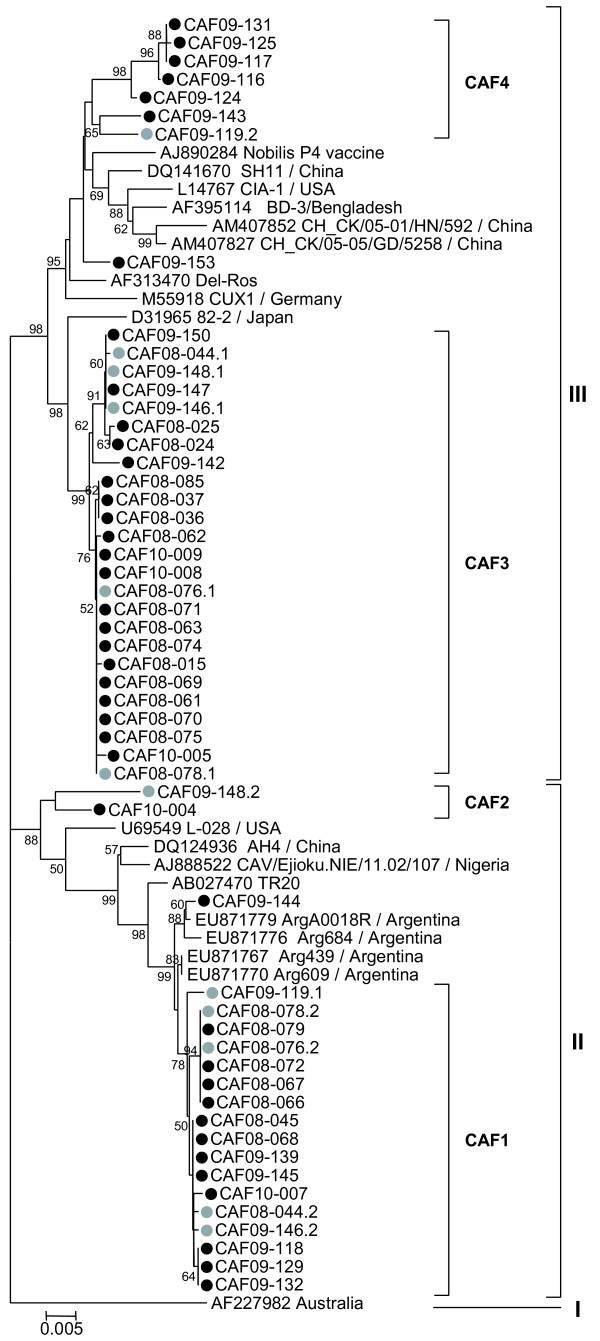
**Detailed phylogenetic analyses of all strains from CAF (53 strains).** Fewer reference strains from GenBank were used in comparison to Figure 2 due to figure size restrictions. Symbols are as in Figure 2. Only bootstrap values ≥ 50% are shown.

Sequencing of the VP1 coding region was attempted for all CAV positive samples but only 228 sequences of at least 1281 nucleotides from the 433 positive samples were obtained. The phylogeny of these 228 VP1 gene sequences (1281 bp) revealed several clusters within group II or III in both countries (Figure 2). The 53 VP1 sequences from 20 chicken flocks and 3 live bird markets in Bangui formed 4 groups (CAF1 to CAF4; Figures 2 and 3). CAF1 strains were very similar to each other (maximal Kimura distance mKd 0.5%) and 1 strain CAF09-144 clustered outside CAF1 group (mKd 0.9% to CAF1). The CAF1 strains were most closely related to strains from Argentina, 2007–2008 (Figure 3). The CAF2 group consisted of only 2 strains (mKd 2.1%) that formed an isolated cluster within group II (Figure 3). Twenty-five strains formed the CAF3 group (mKd 0.8%; Figure 3). The CAF4 group contained 8 strains and was as diverse as CAF2 (mKd 2.1%), but the clustering of CAF09-153 was uncertain as shown by the discrepancies in the trees of Figures 2 and 3. All groups contained strains from farms and live bird markets, except CAF2 which contained strains from markets only. All strains from 2008 clustered in CAF1 and CAF3; 2009 strains were found in all groups and 2010 strains in CAF1 to 3.

**Figure 4:**
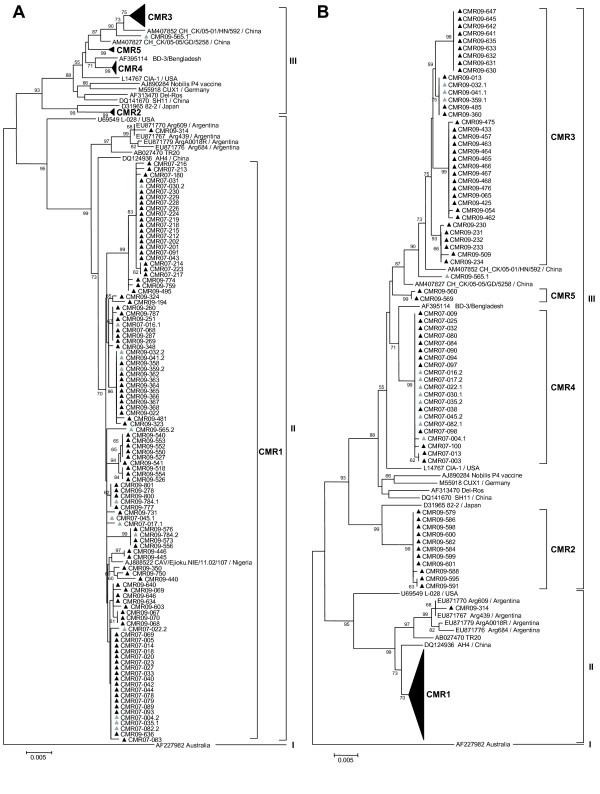
**Detailed phylogenetic analyses of CMR1 (A) and CMR 2 to 5 (B) strains from Cameroon (175 strains).** Fewer reference strains from GenBank were used in comparison to Figure 2 due to figure size restrictions. Symbols are as in Figure 2. Only bootstrap values ≥ 50% are shown.

One hundred seventy-five sequences from chickens in Cameroon clustered in 5 main groups (CMR1 to CMR5; Figures 2 and 4). CMR1 cluster included 104 strains from both 2007 and 2009 (mKd 1.4%), and a Nigerian strain CAV/Ejioku.NIE/11.02/107 (Figure 4A). CMR2 contained 11 strains from 2009 (mKd 0.2%; Figure 4B) and was most closely related to a Japanese strain and to the CAF3 cluster (Figure 2). The CMR3 group included 35 strains from 2009 (mKd 0.9%) and was most closely related to a Chinese strain CH_CK/05-01/HN/592 (Figure 4B). In addition, 1 strain CMR09-565.1 clustered outside CMR3 group (mKd to CMR3 of 1.3%; Figure 2). CMR4 cluster contained 21 very similar strains from 2007 only (mKd 0.2%). Two strains from 2009 clustered in CMR5 (mKd 0.1%; Figure 4B), and one strain (CMR09-314) clustered with Argentinean strains but did not intermingle with CAF1 strains (Figures 2 and 4B).

### Mixed infections

After cloning, sequences of the clones were compared to the original electropherograms of the (uncloned) sample showing signs of mixed infection. The electropherogram of the original sample and the clones matched in all cases, confirming in two independent sequencing experiments that the mixed infections were not due to contamination during the amplification steps.

In Central African Republic, 6 flocks out of 20 from which CAV sequences were obtained, contained strains of 2 different genotypes (Table 3), either CAF1 and CAF3, or CAF1 and CAF4 strains. CAF1 and CAF4 strains were also both found in 2 markets. In addition, analyses of VP1 clones from samples with ambiguous nucleotides revealed mixed infections in 6 individual samples with the above combinations of genotypes (CAF1 and CAF3, CAF1 and CAF4) as well as CAF2 and CAF3 (Figure 3). In 3 of these cases, both parental strains were also found in other samples from the same farm or market (Table 3).

**Table 3:** Mixed infections in flocks, markets and in individual samples from Central African Republic

**Flock**	**Sample**	**Clusters**
Flock 1	CAF08-044	CAF1 + CAF3
	CAF08-045	CAF1
Flock 2	CAF08-068	CAF1
	CAF08-069	CAF3
	CAF08-070	CAF3
	CAF08-071	CAF3
Flock 3	CAF08-072	CAF1
	CAF08-074	CAF3
	CAF08-075	CAF3
Flock 4	CAF08-076	CAF1 + CAF3
	CAF08-078	CAF1 + CAF3
	CAF08-079	CAF1
Flock 5	CAF09-131	CAF4
	CAF09-132	CAF1
Flock 6	CAF09-144	-
	CAF09-145	CAF1
	CAF09-146	CAF1 + CAF3
	CAF09-147	CAF3
Market 1	CAF09-119	CAF1 + CAF4
	CAF09-116	CAF4
	CAF09-117	CAF4
	CAF09-118	CAF1
Market 2	CAF09-142	CAF1
	CAF09-143	CAF4
Market 3	CAF09-148	CAF2 + CAF3

Similarly, CAV VP1 sequences in 13 samples from Cameroon out of 35 showing ambiguous nucleotides were cloned. Analyses of clones showed that these samples contained more than one CAV genotype, suggesting mixed infections in 2007 with CMR1 and CMR4 and in 2009 with CMR1 and CMR3 strains or with 2 distinct CMR1 strains (CMR09-784.1 and CMR09-784.2) differing by 7 nucleotides (Figure 4).

### Phylogenetic analyses of amino acid sequences

**Figure 5:**
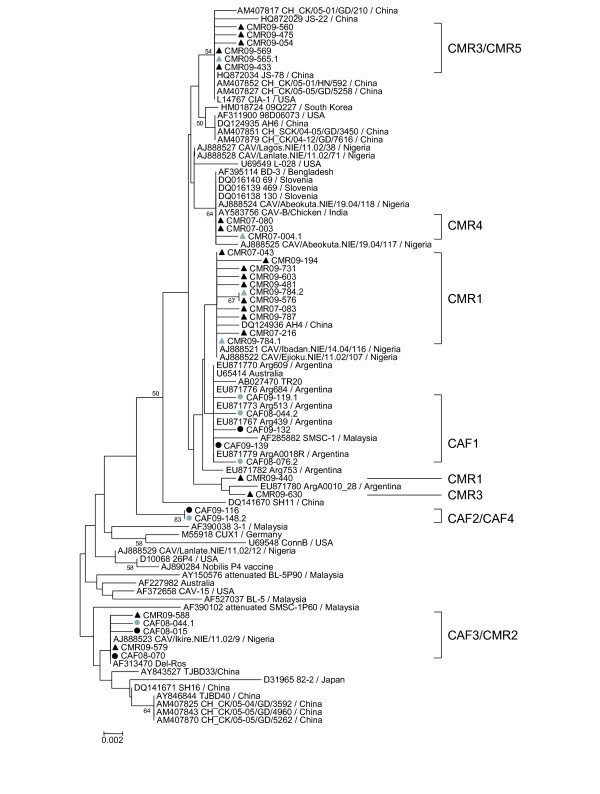
**Phylogenetic analysis of the predicted amino acid sequences.** Representative strains from each CAF and CMR clusters were used and all strains with amino acid substitutions compared to a reference strain within the group were included. Symbols are as in Figure 2. Only bootstrap values ≥ 50% are shown.

At the amino acid level, the groups I to III defined at the nucleotide level were less clearly distinguishable (Figure 5). Nevertheless most of the CAF/CMR strains still clustered with sequences of their own nucleotide sequence group sharing specific amino acid sequences (Table 4), except for CMR09-440 (CMR1) and CMR09-630 (CMR3), both of which clustered together with an Argentinian strain (ArgA0010_28), as a result of Q139K and Q144E mutations located in the hypervariable region. CAF2 and CAF4 sequences, CAF3 and CMR2, and CMR3 and CMR5 sequences were identical at the amino acid level as a result of synonymous mutations (Table 4). All CAF/CMR clusters shared amino acids with published sequences except the CAF2/CAF4 group that had a unique amino acid pattern (Table 4, Figure 5).

**Table 4:** Amino acid patterns in VP1 protein of the African genomic groups

**Cluster**	**22**	**75**	**97**	**139**	**144**	**287**	**290**	**370**	**413**
CAF1	H	I	L	Q	Q	T	P	S	A
CAF2/CAF4	H	V	M	Q	Q	S	A	G	S
CAF3/CMR2	H	V	M	K	E	S	A	G	S
CMR1	H	I	L	Q/K	Q/E	T	A	S	A
CMR3/CMR5	N	I	L	Q/K	Q/E	A	A	S	A
CMR4	H	I	L	Q	Q	A	A	T	A

Sixteen unique amino acid substitutions were observed in the strains from Central African Republic and Cameroon (R6L, R9K, F77L, G74E, S178P, G219E, S229Y, I285V, S300N, M311I, K341R, Q351H, T361A, D366E, G398D, G426S) and 3 substitutions were found in 3 to 6 strains (L190M, T215A, V385I).

## Discussion

The overall flock seroprevalence of 36.7% in CAF appeared to be lower than in most other developing countries. In Nigeria, the only African country from where such data are available, a flock seroprevalence of 55% was reported in 20 flocks including broilers, pullets, cockerels and layers in the main hub of the poultry industry in the Southwest of the country [27]. Even a seroprevalence of 89% was reported later in chicken flocks of 4 to 12 months of age [24]. Also countries such as China [28], Malaysia [29], Japan [4], India [30] and Hungary [31] reported flock seroprevalences ranging from 66 to 100%. Normally the seroconversion is not homogeneous within a flock and tends to increase with age, complicating comparisons between studies [5,32]. For instance broiler flocks of 3 to 10 weeks have a similar seroprevalence in Nigeria (40%) [27] and in CAF (34%) and the seroprevalence in the flocks ranges from 20 to 100% in both countries (Figure 1A).

As in Nigeria, chickens are not vaccinated against CAV in Central African Republic. All sampled flocks except 2, were older than 2 to 3 weeks, the age at which the maternal antibodies wane [5,19,33]. Thus the antibodies detected are likely the result of a field infection and are not maternally derived nor vaccine induced antibodies.

Similar overall virus prevalences were found in 2007 and 2009 in Cameroon (39% and 34.9%) and in 2008 in Central African Republic (34.2%). In 2009 and 2010, the prevalence decreased to 14.3% and 10.4% in Central African Republic. This trend to lower incidence rates in Central African Republic may correspond to an improved sensitivity to hygiene due to farmer awareness in the aftermath of the first results of the study. The above values were also surprisingly low compared to other countries such as China (87%) [34], Malaysia (80%) [29] and Argentina (49%) [35] but comparable to Nigeria (41%) [23] for the 2007–2008 period. Although the prevalence may vary with time, this may indicate that the virus is less prevalent in sub-Saharan Africa than in Asia or South America. Nevertheless, prevalence rates of viral nucleic acids and antibodies may be underestimated in particular when the prevalence is low in a flock and/or low numbers of chickens were analyzed per flock. For instance, by analyzing 4 samples per flock, the certainty of detecting antigens or antibodies in a infected flock decreases from 93% when the prevalence is 50%, to 68% when the prevalence is 25%. A lower expected virus prevalence in the digestive tract compared to lymphoid organs [29], the relative young age of the animals, and possibly co-infections with other pathogens may also contribute to underestimate prevalence rates.

CAV DNA could be detected in cloacal samples of a large majority (76.9%) of the seropositive chickens, indicating that these animals may shed the virus despite antibodies (Figure 1B). While antibodies normally develop within 1 to 3 weeks [6,8,12,36], viral particles can be detected in feces up to 5 to 7 weeks post infection [8,12] suggesting that the virus is cleared with considerable delay after the development of virus-neutralizing antibodies. If we assume that most chickens become infected early after the waning of maternal antibodies, persistence of virus may even be longer. This could explain the high prevalence of virus in seroconverted animals. Alternatively co-infections with very virulent IBDV, which is also circulating in West and Central Africa [37-39], may cause a delay in development of anti-CAV antibodies, resulting in CAV persistence and a prolonged viral shedding in feces [20]. Besides the detection of a second round of viral infection cannot be ruled out although the expected excretion period would be short [12].

Although there is no clear geographic clustering of CAV strains worldwide and despite the low mutation rate at the amino acid level [21], most of the strains sequenced in CAF and CMR belonged to 9 distinct clusters at the nucleotide level and were not intermixed with strains from other continent. This suggests multiple introductions of CAV in Central Africa resulting in several clusters that emerged locally and had time to spread and diverge. Normally, chicks used to populate commercial farms in CAF are imported from Europe, but European strains (Germany, Slovenia) available in GenBank are phylogenetically not the closest relatives.

In our study, 6 flocks contained more than 1 CAV strain and 19 samples revealed mixed infections as confirmed by cloning. Similarly, cloning of VP1 sequences from Nigeria [23] and the USA [40] revealed the presence of 2 different strains (belonging to groups II and III) in the same sample. Sequences exhibiting ambiguous nucleotides have also been found in China [34] and Brazil [41] but these were not further investigated. Thus, mixed infection by CAV strains may be a relatively common event, but its impact is currently unknown.

## Conclusions

Despite the low (sero) prevalence found in Central African Republic and Cameroon compared to other countries, several clusters including only African strains were found. This suggests multiple introductions of CAV that spread and diverged locally. Mixed genotype infections together with the observation of CAV DNA in cloacal samples despite antibodies suggest a suboptimal protection by antibodies or virus persistence.

## Methods

### Sample cohorts

In the framework of enhanced laboratory surveillance for avian viruses in the aftermath of avian influenza outbreaks in West and Central Africa, an average of 4 (between 1 and 16) cloacal samples per flock (broilers or layers) in 5 randomly selected farms were collected every month between June 2008 and December 2010 (*n* = 606) in Bangui (CAF). Cloacal swabs (*n* = 198) were also collected in two live bird markets every month between April 2009 and December 2010. In addition, 400 sera from swabbed birds were collected. Whenever recorded (in 73% of the flocks), the age of the animals ranged from 18 to 68 days for broiler flocks and from 8 weeks to 3 years for layer flocks.

In Cameroon, 287 cloacal swabs and 582 pooled tracheal-cloacal swabs from chicken were collected in 2007 (January to June) and 2009 (April to June) respectively in farms only (2007) or in farms and live bird markets (2009) within a 50 km radius around the town of Bamenda, North West Region. The age of the animals was not recorded.

### Enzyme-linked immunosorbent assay (ELISA)

A commercial competitive ELISA kit (FlockCheck® CAV, IDEXX, Hoofddorp, The Netherlands) was used to detect specific antibodies against CAV in chicken sera (*n* = 400) at a 1:10 dilution. Sera samples were collected from birds in farms (*n* = 302) and live bird markets (*n* = 98). Optical density was measured using a Multiskan Ascent reader (Thermo Labsystems, Helsinki, Finland) at 650 nm. Sample to negative (S/N) ratios were calculated for each sample and samples with S/N ratios ≤ 0.60 were considered positive.

### Nucleic acid extraction, polymerase chain reactions

All swabs were discharged in 500 μl of virus transport medium (VTM) containing 2000 U/ml penicillin, 200 mg/ml streptomycin, 2000 U/ml polymyxin B, 250 mg/ml gentamycin, 60 mg/ml ofloxacin, 200 mg/ml sulfamethoxazole and 2.5 mg/ml amphotericin B. Nucleic acids were extracted from 140 μl of VTM using QIAamp Viral RNA Mini Kit (Qiagen, Venlo, The Netherlands). Extracted nucleic acids were screened for CAV DNA in a nested PCR format using previously published primers [11]. The equivalent of 2.5 μl of nucleic acids and 0.5 μl of first round PCR products were used in the first round and nested PCRs respectively. PCR reactions were carried out using the following cycling conditions: initial denaturation at 95°C for 5 min, 40 cycles of amplification at 95°C for 30 s, 54°C (1^st^ round) or 60°C (nested) for 30 s, 72°C for 1 min, and a final extension at 72°C for 10 min. PCR products were visualized by gel electrophoresis.

The certainty *C* of detecting CAV in an infected flock for various flock sizes *N* and various prevalences *P* was calculated according to the formula of Cannon and Roe (1982, cited by [42]): $n=\left\{1\;–\;{\left(1\;–\;\text{C}\right)}^{1/\text{PxNxsens}}\right\}\left\{\text{N}–0.5\left(\text{PxN}–1\right)\right\}$ assuming an average number of *n* = 4 samples collected per flock and a test sensitivity (sens) of 100%.

### Sequencing

A fragment containing the entire VP1 coding region (nucleotides 1 to 1350) of the positive samples was amplified as 3 partially overlapping fragments with a total length of 1389 bp (from nucleotide −33 to nucleotide 1355) using several primer combinations [23]. Sequencing of purified PCR products was performed as previously described [34]. Sequence assembly and analyses were performed using SeqScape version 2.5 (Applied Biosystems, Nieuwerkerk, The Netherlands) and BioEdit [43]. The 5’ end of the VP1 coding region (69 nucleotides) was not reliably sequenced for a few strains. Therefore a shorter fragment (1281 nucleotides) was used in the phylogenetic analyses in order to include as many strains as possible.

### Cloning

Samples exhibiting ambiguous determination of nucleotides at various positions caused by clear double peaks in the electropherogram were further analyzed. The VP1 fragments of 1370 bp were generated starting from the original DNA sample using primers OS1F and S3R7 (1^st^ round PCR; nucleotides −117 to 1355) and S1F and S3R1 (5’-CCCAGTACATSGTGCTGTT-3’) primers (nested PCR; nucleotides −33 to 1336), purified and cloned using the TOPO TA cloning kit (Life Technologies, Merelbeke, Belgium) as described previously [23]. Ten to 24 colonies were selected and inserts were sequenced with M13 primers (Life Technologies). To exclude the possibility of crosscontamination, long amplicons (1370 bp) suitable for cloning were generated twice in two independent experiments (on different days) and were cloned separately (again on different days) for two samples and similar results were obtained. Both experiments gave clones of the same sequences although with different frequencies.

### Phylogenetic analyses

Genetic distances were calculated with MEGA v5.03 [44] according to the Kimura 2-parameters model. Phylogenetic relationships were inferred by comparing the African strains with all CAV DNA sequences available on GenBank (downloaded in February 2012) after removal of short sequences and sequences with insertions or deletions resulting in frame shifts. Datasets were aligned using ClustalW [45]. Trees were calculated with the Neighbour-Joining method, using the Kimura 2-parameters model and 1000 bootstrap replicates for the nucleotide tree and with the Poisson model for the amino acid tree (MEGA v5.03; data not shown). Reference strains from GenBank were selected based on these preliminary analyses. Trees including the selected reference strains from GenBank and all (Figure 2) or a subset (Figure 5) of the sequences generated in this study were calculated using the same parameters as described above. Fewer representative strains from GenBank were used in Figures 3 and 4 due to figure size restrictions. All gene sequences can be found under accession numbers [EMBL: HE662876 to HE663056 and HE686970 to HE687016]. Strains were designated using the following nomenclature: 3-letter country code (CAF = Central African Republic; CMR = Cameroon)_year-sample number (last 3 digits). Sequences from mixed infected samples are named by adding .1 or .2 after the strain name.

## Abbreviations

bp: Base pair(s); CAF: Central African Republic; CAV: Chicken anemia virus; CMR: Cameroon; ELISA: Enzyme-linked immunosorbent assay; IBDV: Infectious bursal disease virus; mKd: Maximal Kimura distance; PCR: Polymerase chain reaction; S/N: Sample to negative; VP1: Viral protein 1; VTM: Virus transport medium.

## Competing interests

The authors declare that they have no competing interests.

## Author’s contributions

CJS, EN, MPO, AL and CPM designed the study. CJS, GFK and BPM carried out the experiments and analyzed the results. CJS drafted the manuscript and AL, EN, MPO and CPM contributed to the discussion and reviewed the manuscript. All authors read and approved the final manuscript.

## References

[B1] Virus taxonomy: release 2009 http://ictvonline.org/virusTaxonomy.asp?version=2009

[B2] Yuasa N Taniguchi T Yoshida I Isolation and some characteristics of an agent inducing anemia in chicks Avian Dis 1979 23 366 385 10.2307/1589567

[B3] Toro H Ewald S Hoerr FJ Serological evidence of chicken infectious anemia virus in the United States at least since 1959 Avian Dis 2006 50 124 126 10.1637/7442-092205R.1 16617995

[B4] Farkas T Maeda K Sugiura H Kai K Hirai K Otsuki K Hayashi T A serological survey of chickens, Japanese quail, pigeons, ducks and crows for antibodies to chicken anaemia virus (CAV) in Japan Avian Pathol 1998 27 316 320 10.1080/03079459808419344 18484006

[B5] McNulty MS Connor TJ McNeilly F Kirkpatrick KS McFerran JB A serological survey of domestic poultry in the United Kingdom for antibody to chicken anaemia agent Avian Pathol 1988 17 315 324 10.1080/03079458808436450 18766689

[B6] Schat KA Saif YM, Barnes HJ, Glisson JR, Fadly AM, McDougald LR, Swayne DE Chicken Infectious Anemia Diseases of Poultry 2003 11 Iowa State University Press, Ames, Iowa 182 202

[B7] Miller MM Schat KA Chicken infectious anemia virus: an example of the ultimate host-parasite relationship Avian Dis 2004 48 734 745 10.1637/7271-090304R 15666854

[B8] Yuasa N Taniguchi T Imada T Hihara H Distribution of chicken anemia agent (CAA) and detection of neutralizing antibody in chicks experimentally inoculated with CAA Natl Inst Anim Health Q (Tokyo) 1983 23 78 81 6677833

[B9] Davidson I Artzi N Shkoda I Lublin A Loeb E Schat KA The contribution of feathers in the spread of chicken anemia virus Virus Res 2008 132 152 159 10.1016/j.virusres.2007.11.012 18177972

[B10] Rosenberger JK Cloud SS The effects of age, route of exposure, and coinfection with infectious bursal disease virus on the pathogenicity and transmissibility of chicken anemia agent (CAA) Avian Dis 1989 33 753 759 10.2307/1591156 2559706

[B11] Cardona CJ Oswald WB Schat KA Distribution of chicken anaemia virus in the reproductive tissues of specific-pathogen-free chickens J Gen Virol 2000 81 2067 2075 10900046 10.1099/0022-1317-81-8-2067

[B12] Hoop RK Persistence and vertical transmission of chicken anaemia agent in experimentally infected laying hens Avian Pathol 1992 21 v493 501 10.1080/03079459208418867 18670964

[B13] Brentano L Lazzarin S Bassi SS Klein TA Schat KA Detection of chicken anemia virus in the gonads and in the progeny of broiler breeder hens with high neutralizing antibody titers Vet Microbiol 2005 105 65 72 10.1016/j.vetmic.2004.09.019 15607085

[B14] Miller MM Ealey KA Oswald WB Schat KA Detection of chicken anemia virus DNA in embryonal tissues and eggshell membranes Avian Dis 2003 47 662 671 10.1637/7007 14562895

[B15] Miller MM Jarosinski KW Schat KA Negative modulation of the chicken infectious anemia virus promoter by COUP-TF1 and an E box-like element at the transcription start site binding deltaEF1 J Gen Virol 2008 89 2998 3003 10.1099/vir.0.2008/003103-0 19008385

[B16] Davidson I Kedem M Borochovitz H Kass N Ayali G Hamzani E Perelman B Smith B Perk S Chicken infectious anemia virus infection in Israeli commercial flocks: virus amplification, clinical signs, performance, and antibody status Avian Dis 2004 48 108 118 10.1637/7072 15077804

[B17] McIlroy SG McNulty MS Bruce DW Smyth JA Goodall EA Alcorn MJ Economic effects of clinical chicken anemia agent infection on profitable broiler production Avian Dis 1992 36 566 574 10.2307/1591750 1417588

[B18] McNulty MS McIlroy SG Bruce DW Todd D Economic effects of subclinical chicken anemia agent infection in broiler chickens Avian Dis 1991 35 263 268 10.2307/1591175 1854312

[B19] Sommer F Cardona C Chicken anemia virus in broilers: dynamics of the infection in two commercial broiler flocks Avian Dis 2003 47 1466 1473 10.1637/7048 14708998

[B20] Imai K Mase M Tsukamoto K Hihara H Yuasa N Persistent infection with chicken anaemia virus and some effects of highly virulent infectious bursal disease virus infection on its persistency Res Vet Sci 1999 67 233 238 10.1053/rvsc.1999.0313 10607503

[B21] Schat KA Chicken anemia virus Curr Top Microbiol Immunol 2009 331 151 183 10.1007/978-3-540-70972-5_10 19230563

[B22] Wicht JD Maharaj SB Chicken anaemia agent in South Africa Vet Rec 1993 133 147 148 8236695 10.1136/vr.133.6.147

[B23] Ducatez MF Owoade AA Abiola JO Muller CP Molecular epidemiology of chicken anemia virus in Nigeria Arch Virol 2006 151 97 111 10.1007/s00705-005-0609-7 16096706

[B24] Emikpe BO Oluwayelu DO Ohore OG Oladele OA Oladokun AT Serological evidence of chicken anaemia virus infection in Nigerian indigenous chickens Onderstepoort J Vet Res 2005 72 101 103 15991709 10.4102/ojvr.v72i1.227

[B25] Oluwayelu DO Todd D Ball NW Scott AN Oladele OA Emikpe BO Fagbohun OA Owoade AA Olaleye OD Isolation and preliminary characterization of chicken anemia virus from chickens in Nigeria Avian Dis 2005 49 446 450 10.1637/7339-020705R.1 16252505

[B26] Oluwayelu DO Todd D Olaleye OD Sequence and phylogenetic analysis of chicken anaemia virus obtained from backyard and commercial chickens in Nigeria Onderstepoort J Vet Res 2008 75 353 357 19294991 10.4102/ojvr.v75i4.111

[B27] Owoade AA Oluwayelu DO Fagbohun OA Ammerlaan W Mulders MN Muller CP Serologic evidence of chicken infectious anemia in commercial chicken flocks in southwest Nigeria Avian Dis 2004 48 202 205 10.1637/7075 15077816

[B28] Zhou W Yang B Shen B Han S Zhou J A serologic survey of antibody against chicken infectious anemia virus by indirect immunofluorescent assay in domestic poultry in China Avian Dis 1996 40 358 360 10.2307/1592232 8790886

[B29] Hailemariam Z Omar AR Hair-Bejo M Giap TC Detection and characterization of chicken anemia virus from commercial broiler breeder chickens Virol J 2008 5 128 10.1186/1743-422X-5-128 18954433 PMC2605446

[B30] Bhatt P Shukla SK Mahendran M Dhama K Chawak MM Kataria JM Prevalence of Chicken Infectious Anaemia Virus (CIAV) in commercial poultry flocks of Northern India: a serological survey Transbound Emerg Dis 2011 58 458 460 10.1111/j.1865-1682.2011.01215.x 21414182

[B31] Dren C Farkas T Nemeth I Serological survey on the prevalence of chicken anaemia virus infection in Hungarian chicken flocks Vet Microbiol 1996 50 7 16 10.1016/0378-1135(96)00002-8 8810003

[B32] De Herdt P Van den Bosch G Ducatelle R Uyttebroek E Schrier C Epidemiology and significance of chicken infectious anemia virus infections in broilers and broiler parents under nonvaccinated European circumstances Avian Dis 2001 45 706 708 10.2307/1592916 11569748

[B33] Otaki Y Saito K Tajima M Nomura Y Persistence of maternal antibody to chicken anaemia agent and its effect on the susceptibility of young chickens Avian Pathol 1992 21 147 151 10.1080/03079459208418828 18670925

[B34] Ducatez MF Chen H Guan Y Muller CP Molecular epidemiology of chicken anemia virus (CAV) in southeastern Chinese live birds markets Avian Dis 2008 52 68 73 10.1637/8049-070407-Reg 18459299

[B35] Craig MI Rimondi A Delamer M Sansalone P Konig G Vagnozzi A Pereda A Molecular characterization of chicken infectious anemia virus circulating in Argentina during 2007 Avian Dis 2009 53 331 335 10.1637/8478-100808-Reg.1 19848068

[B36] van Santen VL Joiner KS Murray C Petrenko N Hoerr FJ Toro H Pathogenesis of chicken anemia virus: comparison of the oral and the intramuscular routes of infection Avian Dis 2004 48 494 504 10.1637/7155-010904R 15529971

[B37] Courtecuisse C Japiot F Bloch N Diallo I Serological survey on newcastle and gumboro diseases, pasteurellosis and pullorosis in local hens in Niger Rev Elev Med Vet Pays Trop 1990 43 27 29 2175925

[B38] Durojaiye OA Kwenkam P A preliminary note on the prevalence of infectious bursal disease of poultry in Cameroon Rev Elev Med Vet Pays Trop 1990 43 439 440 1966758

[B39] Owoade AA Mulders MN Kohnen J Ammerlaan W Muller CP High sequence diversity in infectious bursal disease virus serotype 1 in poultry and turkey suggests West-African origin of very virulent strains Arch Virol 2004 149 653 672 10.1007/s00705-003-0270-y 15045556

[B40] van Santen VL Li L Hoerr FJ Lauerman LH Genetic characterization of chicken anemia virus from commercial broiler chickens in Alabama Avian Dis 2001 45 373 388 10.2307/1592977 11417817

[B41] Simionatto S Lima-Rosa CA Binneck E Ravazzolo AP Canal CW Characterization and phylogenetic analysis of Brazilian chicken anaemia virus Virus Genes 2006 33 5 10 10.1007/s11262-005-0033-9 16791412

[B42] De Wit JJ Detection of infectious bronchitis virus Avian Pathol 2000 29 71 93 10.1080/03079450094108 19184793

[B43] Hall TA BioEdit: a user-friendly biological sequence alignment editor and analysis program for Windows 95/98/NT Nucleic Acids Symp Ser 1999 41 95 98

[B44] Tamura K Peterson D Peterson N Stecher G Nei M Kumar S MEGA5: molecular evolutionary genetics analysis using maximum likelihood, evolutionary distance, and maximum parsimony methods Mol Biol Evol 2011 28 2731 2739 10.1093/molbev/msr121 21546353 PMC3203626

[B45] Thompson JD Higgins DG Gibson TJ CLUSTAL W: improving the sensitivity of progressive multiple sequence alignment through sequence weighting, position-specific gap penalties and weight matrix choice Nucleic Acids Res 1994 22 4673 4680 10.1093/nar/22.22.4673 7984417 PMC308517

